# Supplementary figures and images for: Genome-wide identification and expression profile analysis of the Hsp20 gene family in Barley (Hordeum vulgare L.)
Source: PeerJ. 2019 May 3;7:e6832. doi: 10.7717/peerj.6832 (PMC6501772; doi:10.7717/peerj.6832)

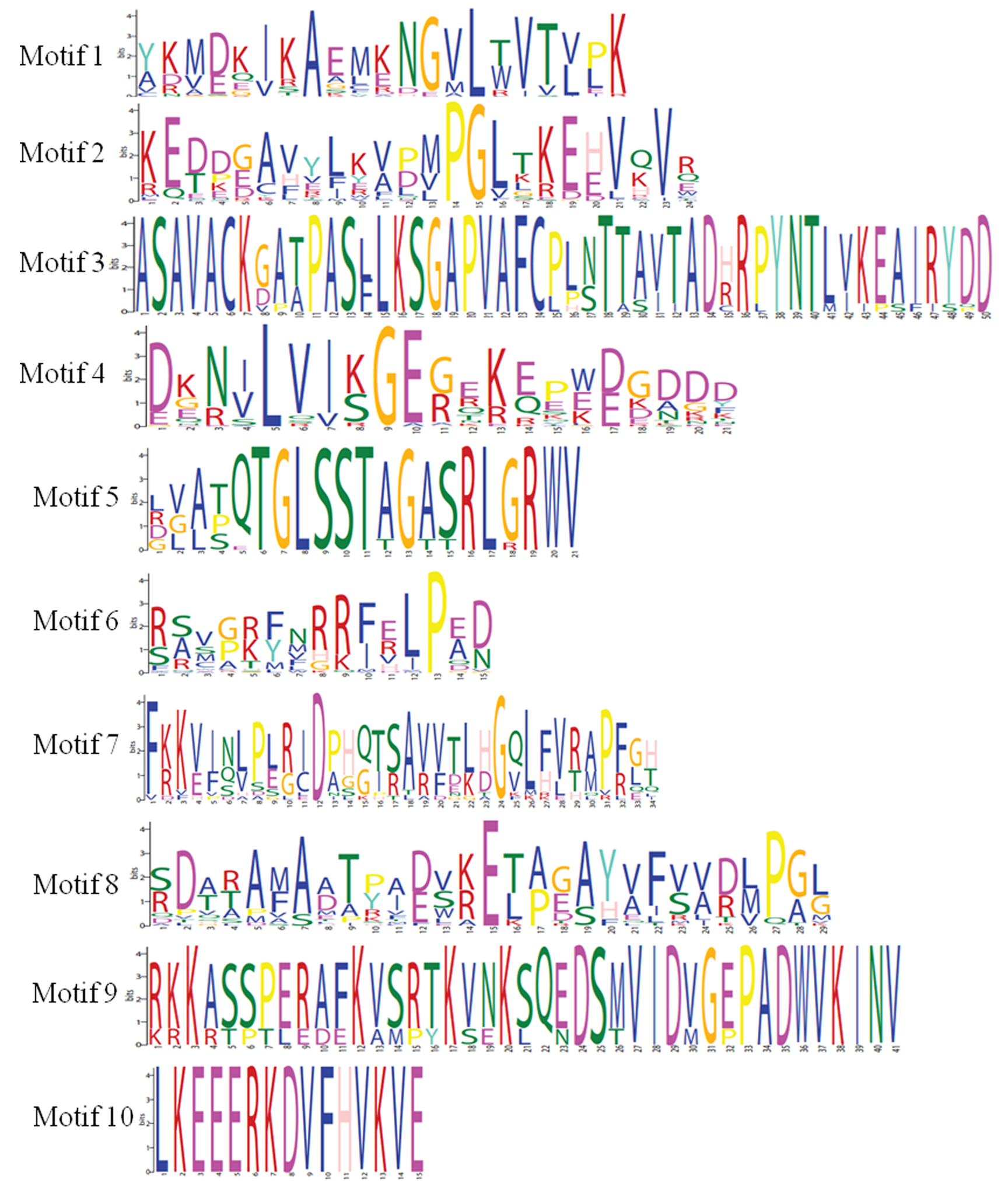

Supplement: Supplemental Information 1 [file peerj-07-6832-s001.png]

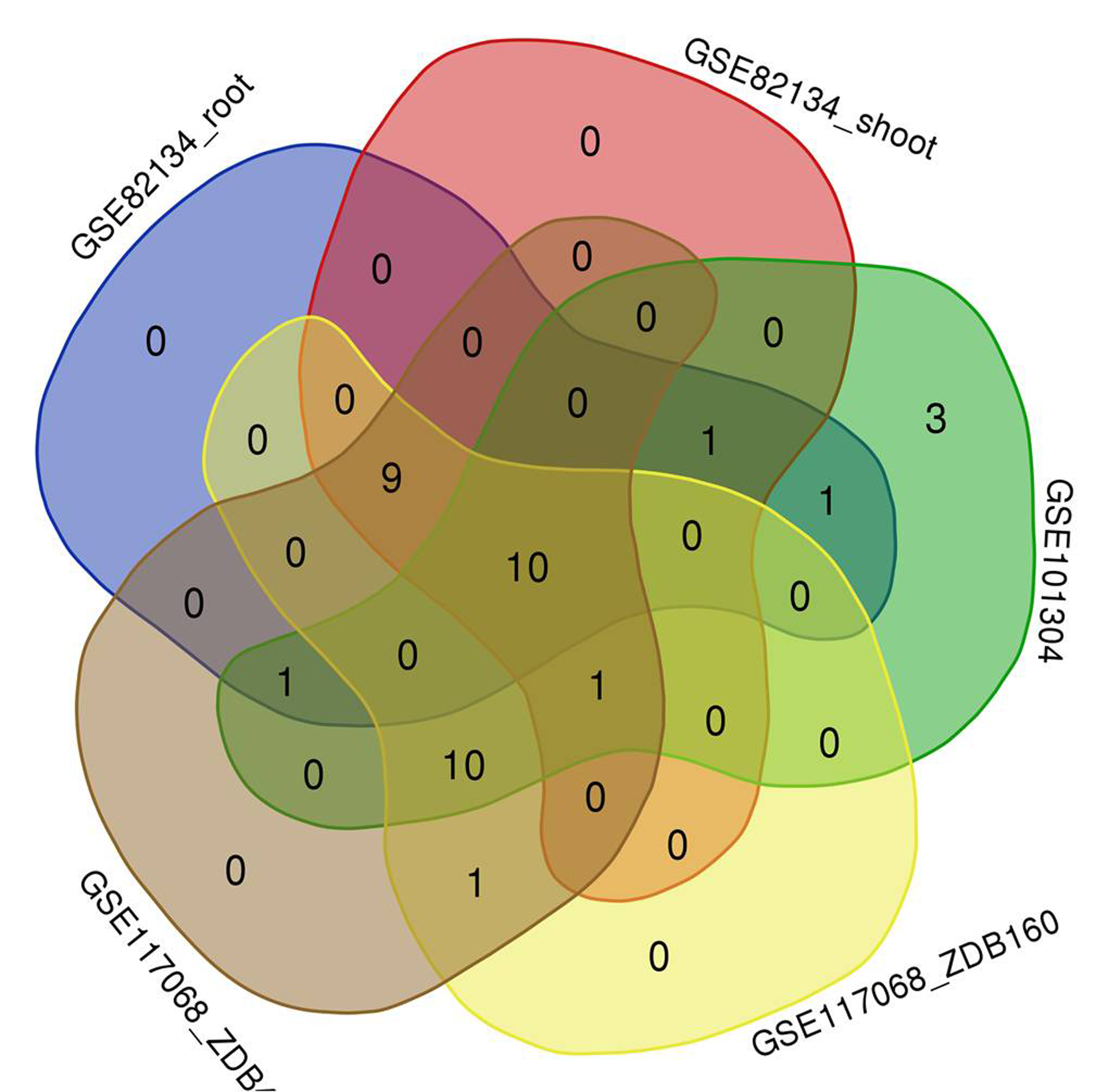

Supplement: Supplemental Information 2 [file peerj-07-6832-s002.png]
