# Supplementary material for: Genome-wide identification and expression profile analysis of the Hsp20 gene family in Barley (Hordeum vulgare L.)
Source: PeerJ. 2019 May 3;7:e6832. doi: 10.7717/peerj.6832 (PMC6501772; doi:10.7717/peerj.6832)
Supplement: Supplemental Information 4 [file peerj-07-6832-s004.docx]

**Table S2** The location and sequence of motifs in barley Hsp20s.

Motif 1 located in conserved region I of ACD domain of barley Hsp20s.


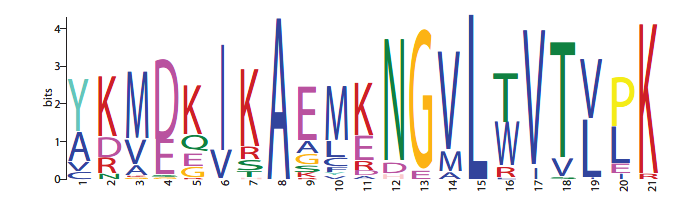


| Name | Start | *p*-value |  | Sites |  |
| --- | --- | --- | --- | --- | --- |
| HORVU7Hr1G036540.6 | 107 | 2.05e-27 | **NRRIELPADA** | **YKMDKIKAEMKNGVLWVTLLK** | **LKEEERKDVF** |
| HORVU7Hr1G036540.3 | 223 | 2.05e-27 | **NRRIELPADA** | **YKMDKIKAEMKNGVLWVTLLK** | **LKEEERKDVF** |
| HORVU7Hr1G036540.5 | 174 | 2.05e-27 | **NRRIELPADA** | **YKMDKIKAEMKNGVLWVTLLK** | **LKEEERKDVF** |
| HORVU7Hr1G036470.21 | 183 | 2.05e-27 | **NRRIELPADA** | **YKMDKIKAEMKNGVLWVTLLK** | **LKEEERKDVF** |
| HORVU7Hr1G036470.20 | 187 | 2.05e-27 | **NRRIELPADA** | **YKMDKIKAEMKNGVLWVTLLK** | **LKEEERKDVF** |
| HORVU7Hr1G036570.3 | 181 | 2.05e-27 | **NRRIELPADA** | **YKMDKIKAEMKNGVLWVTLLK** | **LKEEERKDVF** |
| HORVU7Hr1G036570.2 | 186 | 2.05e-27 | **NRRIELPADA** | **YKMDKIKAEMKNGVLWVTLLK** | **LKEEERKDVF** |
| HORVU7Hr1G036570.4 | 166 | 2.05e-27 | **NRRIELPADA** | **YKMDKIKAEMKNGVLWVTLLK** | **LKEEERKDVF** |
| HORVU7Hr1G036500.7 | 169 | 2.05e-27 | **NRRIELPADA** | **YKMDKIKAEMKNGVLWVTLLK** | **LKEEERKDVF** |
| HORVU7Hr1G036500.2 | 163 | 2.05e-27 | **NRRIELPADA** | **YKMDKIKAEMKNGVLWVTLLK** | **LKEEERKDVF** |
| HORVU7Hr1G036470.12 | 168 | 4.09e-27 | **NHRIEIPADA** | **YKMDKIKAEMKNGMLWVTLLK** | **L** |
| HORVU7Hr1G036470.2 | 190 | 4.09e-27 | **NHRIEIPADA** | **YKMDKIKAEMKNGMLWVTLLK** | **LKEEERKDVF** |
| HORVU7Hr1G036470.6 | 179 | 4.09e-27 | **NHRIEIPADA** | **YKMDKIKAEMKNGMLWVTLLK** | **LKEEERKDVF** |
| HORVU7Hr1G036470.14 | 146 | 4.09e-27 | **NHRIEIPADA** | **YKMDKIKAEMKNGMLWVTLLK** |  |
| HORVU7Hr1G036470.11 | 163 | 4.09e-27 | **NHRIEIPADA** | **YKMDKIKAEMKNGMLWVTLLK** | **LXXXXXXXXX** |
| HORVU7Hr1G036470.10 | 206 | 4.09e-27 | **NHRIEIPADA** | **YKMDKIKAEMKNGMLWVTLLK** | **LKEEERKDVF** |
| HORVU7Hr1G036470.9 | 178 | 4.09e-27 | **NHRIEIPADA** | **YKMDKIKAEMKNGMLWVTLLK** | **LKEEERKDVF** |
| HORVU7Hr1G036470.3 | 207 | 4.09e-27 | **NHRIEIPADA** | **YKMDKIKAEMKNGMLWVTLLK** | **LKEEERKDVF** |
| HORVU7Hr1G036540.2 | 105 | 9.49e-27 | **NHRIEIPADA** | **YKMDKIKAEMKNGVLWVTLIK** | **LKEEERKDVF** |
| HORVU7Hr1G036540.1 | 191 | 9.49e-27 | **NHRIEIPADA** | **YKMDKIKAEMKNGVLWVTLIK** | **LKEEERKDVF** |
| HORVU7Hr1G036540.4 | 167 | 9.49e-27 | **NHRIEIPADA** | **YKMDKIKAEMKNGVLWVTLIK** | **LKEEERKDVF** |
| HORVU6Hr1G077710.3 | 240 | 2.54e-22 | **SGRLELTGDV** | **YRMDQIKAEMKNGVLRVVVPK** | **VEKRTDVYEV** |
| HORVU6Hr1G077710.5 | 99 | 2.54e-22 | **SGRLELTGDV** | **YRMDQIKAEMKNGVLRVVVPK** | **VEKRTDVYEV** |
| HORVU6Hr1G077710.2 | 212 | 2.54e-22 | **SGRLELTGDV** | **YRMDQIKAEMKNGVLRVVVPK** | **VEKRTDVYEV** |
| HORVU6Hr1G077710.4 | 134 | 2.54e-22 | **SGRLELTGDV** | **YRMDQIKAEMKNGVLRVVVPK** | **VEKRTDVYEV** |
| HORVU6Hr1G077710.6 | 171 | 2.54e-22 | **SGRLELTGDV** | **YRMDQIKAEMKNGVLRVVVPK** | **VEKRTDVYEV** |
| HORVU4Hr1G060720.1 | 115 | 1.39e-19 | **FLRRFRLPEN** | **AKAEQVKASMENGVLTVTVPK** | **EEAKNPEVKA** |
| HORVU4Hr1G060720.2 | 123 | 1.39e-19 | **FLRRFRLPEN** | **AKAEQVKASMENGVLTVTVPK** | **EEAKNPEVKA** |
| HORVU3Hr1G020520.4 | 125 | 1.87e-19 | **MMRKFVLPEN** | **ADMEKISAACRNGVLTVTVEK** | **LPPPEPKKPK** |
| HORVU3Hr1G020520.6 | 180 | 1.87e-19 | **MMRKFVLPEN** | **ADMEKISAACRNGVLTVTVEK** | **LPPPEPKKPK** |
| HORVU4Hr1G060760.1 | 115 | 2.06e-19 | **FLRRFRLPDN** | **ARAEEIKAAMENGVLTVTVPK** | **AEAKKPDVKP** |
| HORVU3Hr1G007380.1 | 160 | 9.32e-19 | **FVRRFRLPED** | **AKVEEVKAGLENGVLTVTVPK** | **AEVKKPEVKA** |
| HORVU3Hr1G006530.1 | 175 | 9.32e-19 | **FVRRFRLPED** | **AKVEEVKAGLENGVLTVTVPK** | **AEVKKPEVKA** |
| HORVU2Hr1G120170.1 | 213 | 9.32e-19 | **FVRRFRLPED** | **AKVEEVKAGLENGVLTVTVPK** | **AQVKKPEVKA** |
| HORVU3Hr1G007500.1 | 168 | 9.32e-19 | **FVRRFRLPED** | **AKVEEVKAGLENGVLTVTVPK** | **TEVKKPEVKA** |
| HORVU3Hr1G007500.2 | 168 | 9.32e-19 | **FVRRFRLPED** | **AKVEEVKAGLENGVLTVTVPK** | **AEVKKPEVKA** |
| HORVU3Hr1G007500.4 | 181 | 9.32e-19 | **FVRRFRLPED** | **AKVEEVKAGLENGVLTVTVPK** | **AEVKKPEVKA** |
| HORVU3Hr1G020490.2 | 152 | 4.69e-18 | **LMRKFVLPEN** | **ADMEKISAACRDGVLTVTVEK** | **LPPPEPKKPK** |
| HORVU3Hr1G020490.3 | 153 | 4.69e-18 | **LMRKFVLPEN** | **ADMEKISAACRDGVLTVTVEK** | **LPPPEPKKPK** |
| HORVU3Hr1G020520.2 | 154 | 4.69e-18 | **MMRKFVLPEN** | **ADMEKISAACRDGVLTVTVEK** | **LPPPEPKKPK** |
| HORVU0Hr1G020420.2 | 100 | 4.60e-17 | **FAREVALPEH** | **VRVDQIRASVDNGVLTVVVPK** | **EPAPARPRTR** |
| HORVU0Hr1G020420.1 | 133 | 4.60e-17 | **FAREVALPEH** | **VRVDQIRASVDNGVLTVVVPK** | **EPAPARPRTR** |
| HORVU4Hr1G002290.4 | 46 | 1.66e-16 | **FQKDLQLPSD** | **CNVDGIRAKFENEALTITLPK** | **KHPSPQQAAP** |
| HORVU4Hr1G002290.3 | 106 | 1.66e-16 | **FQKDLQLPSD** | **CNVDGIRAKFENEALTITLPK** | **KHPSPQQAAP** |
| HORVU4Hr1G002290.2 | 75 | 1.66e-16 | **FQKDLQLPSD** | **CNVDGIRAKFENEALTITLPK** | **KHPSPQQAAP** |
| HORVU2Hr1G046370.4 | 110 | 1.12e-15 | **FARKFRLPGM** | **VDADGITAEYAHGVLTVTVPR** | **MHNRARPMVN** |
| HORVU2Hr1G046370.3 | 110 | 1.12e-15 | **FARKFRLPGM** | **VDADGITAEYAHGVLTVTVPR** | **MHNRARPMVN** |
| HORVU4Hr1G063350.1 | 271 | 1.51e-15 | **YDMRLALPDE** | **CDKSQVRAELKNGVLLVSVPK** | **RETERKVIDV** |
| HORVU4Hr1G015170.1 | 181 | 2.19e-15 | **FWRRFRMPAG** | **ADVERVTARLEDGVLTVTVPK** | **IAEHQRREPR** |
| HORVU6Hr1G015130.1 | 142 | 2.60e-14 | **RTVRLPPNAD** | **VDGGGVHAALDNGVLTITIPK** | **DDGKKAYGRI** |

Motif 2 located between conserved region I and conserved region II of ACD domain of HvHsp20s.


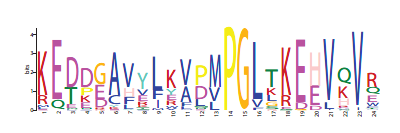


| Name | Start | *p*-value |  | Sites |  |
| --- | --- | --- | --- | --- | --- |
| HORVU7Hr1G036470.12 | 106 | 1.39e-28 | **GASRLGRWVT** | **KEDDGAVYLKVPMPGLTKEHVQVR** | **ADKNILVIKG** |
| HORVU7Hr1G036470.2 | 128 | 1.39e-28 | **GASRLGRWVA** | **KEDDGAVYLKVPMPGLTKEHVQVR** | **ADKNILVIKG** |
| HORVU7Hr1G036470.14 | 84 | 1.39e-28 | **GASRLGRWVA** | **KEDDGAVYLKVPMPGLTKEHVQVR** | **ADKNILVIKG** |
| HORVU7Hr1G036470.11 | 101 | 1.39e-28 | **GASRLGRWVA** | **KEDDGAVYLKVPMPGLTKEHVQVR** | **ADKNILVIKG** |
| HORVU7Hr1G036470.3 | 145 | 1.39e-28 | **GASRLGRWVA** | **KEDDGAVYLKVPMPGLTKEHVQVR** | **ADKNILVIKG** |
| HORVU7Hr1G036570.3 | 119 | 1.39e-28 | **GTSRLGRWVT** | **KEDDGAVYLKVPMPGLTKEHVQVR** | **ADKNILVIKG** |
| HORVU7Hr1G036570.2 | 124 | 1.39e-28 | **GTSRLGRWVT** | **KEDDGAVYLKVPMPGLTKEHVQVR** | **ADKNILVIKG** |
| HORVU7Hr1G036570.4 | 104 | 1.39e-28 | **GTSRLGRWVT** | **KEDDGAVYLKVPMPGLTKEHVQVR** | **ADKNILVIKG** |
| HORVU7Hr1G036500.2 | 101 | 1.39e-28 | **GASRLGRWVA** | **KEDDGAVYLKVPMPGLTKEHVQVR** | **ADKNILVIKG** |
| HORVU7Hr1G036540.6 | 45 | 3.40e-27 | **GASRLGRWVA** | **KEDDGAVYLEVPMPGLTKEHVQVR** | **ADKNILVIKG** |
| HORVU7Hr1G036540.3 | 161 | 3.40e-27 | **GASRLGRWVA** | **KEDDGAVYLEVPMPGLTKEHVQVR** | **ADKNILVIKG** |
| HORVU7Hr1G036540.5 | 112 | 3.40e-27 | **GASRLGRWVA** | **KEDDGAVYLEVPMPGLTKEHVQVR** | **ADKNILVIKG** |
| HORVU7Hr1G036470.10 | 144 | 3.40e-27 | **GASRLRRWVA** | **KEDDGAVYLEVPMPGLTKEHVQVR** | **ADKNILVIKG** |
| HORVU7Hr1G036470.9 | 116 | 3.40e-27 | **GASRLRRWVA** | **KEDDGAVYLEVPMPGLTKEHVQVR** | **ADKNILVIKG** |
| HORVU7Hr1G036540.2 | 43 | 9.92e-27 | **GATRLGRWVA** | **KEDDGAVYLKLPMPGLTKEHVQVR** | **ADKNILVIKG** |
| HORVU7Hr1G036540.1 | 129 | 9.92e-27 | **GATRLGRWVA** | **KEDDGAVYLKLPMPGLTKEHVQVR** | **ADKNILVIKG** |
| HORVU7Hr1G036540.4 | 105 | 9.92e-27 | **GATRLGRWVA** | **KEDDGAVYLKLPMPGLTKEHVQVR** | **ADKNILVIKG** |
| HORVU7Hr1G036470.21 | 121 | 1.49e-26 | **GASRLGRWVT** | **KEDDGAVYLKVPMPGLTKDHVQVC** | **ADKNILVIKG** |
| HORVU7Hr1G036470.6 | 117 | 1.49e-26 | **GASRLGRWVT** | **KEDDGAVYLKVPMPGLTKDHVQVC** | **ADKNILVIKG** |
| HORVU7Hr1G036470.20 | 125 | 1.49e-26 | **GASRLGRWVT** | **KEDDGAVYLKVPMPGLTKDHVQVC** | **ADKNILVIKG** |
| HORVU7Hr1G036500.7 | 107 | 1.20e-25 | **GASRLGRWVA** | **KEDDGAVYLEMPMPGLTKEHVQVR** | **ADKNILVIKG** |
| HORVU7Hr1G036500.5 | 129 | 1.71e-25 | **GASRLGRWVA** | **KEEDGAVYLEVPMPGLTKEHVQVR** | **AGKNILVIKG** |
| HORVU6Hr1G077710.3 | 179 | 3.40e-21 | **AAPLRLGWNA** | **KEDEDALRLRVDMPGLGKEHVKVW** | **AEQNSLVIKG** |
| HORVU6Hr1G077710.2 | 151 | 3.40e-21 | **AAPLRLGWNA** | **KEDEDALRLRVDMPGLGKEHVKVW** | **AEQNSLVIKG** |
| HORVU6Hr1G077710.4 | 73 | 3.40e-21 | **AAPLRLGWNA** | **KEDEDALRLRVDMPGLGKEHVKVW** | **AEQNSLVIKG** |
| HORVU6Hr1G077710.6 | 110 | 3.40e-21 | **AAPLRLGWNA** | **KEDEDALRLRVDMPGLGKEHVKVW** | **AEQNSLVIKG** |
| HORVU4Hr1G060720.2 | 56 | 2.54e-20 | **AAFAGARIDW** | **KETPEAHVFKADVPGLKKEEVKVE** | **VEDGNILQIS** |
| HORVU6Hr1G077710.5 | 38 | 1.79e-19 | **ASSRIPLVRA** | **MENEDALRLRVDMPGLGKEHVKVW** | **AEQNSLVIKG** |
| HORVU4Hr1G060760.1 | 48 | 2.15e-19 | **AAFAGARIDW** | **KETPDAHVFKADVPGLRKEEVKVE** | **VDDGNVLQIS** |
| HORVU3Hr1G007380.1 | 93 | 2.36e-19 | **AAFANARMDW** | **KETPEAHVFKADLPGVKKEEVKVE** | **VEDGNVLVVS** |
| HORVU3Hr1G006530.1 | 108 | 2.36e-19 | **AAFANARMDW** | **KETPEAHVFKADLPGVKKEEVKVE** | **VEDGNVLVVS** |
| HORVU2Hr1G120170.1 | 146 | 2.36e-19 | **AAFASARVDW** | **KETPEAHVFKADLPGVKKEEVKVE** | **VEDGNVLVVS** |
| HORVU3Hr1G007500.1 | 101 | 2.36e-19 | **AAFANARMDW** | **KETPEAHVFKADLPGVKKEEVKVE** | **VEDGNVLVVS** |
| HORVU3Hr1G007500.2 | 101 | 2.36e-19 | **AAFANARMDW** | **KETPEAHVFKADLPGVKKEEVKVE** | **VEDGNVLVVS** |
| HORVU3Hr1G007500.4 | 114 | 2.36e-19 | **AAFANARVDW** | **KETPEAHVFKADLPGVKKEEVKVE** | **VEDGNVLVVS** |
| HORVU6Hr1G070230.11 | 46 | 2.36e-19 | **EPADWVKINV** | **RQTKECFEIYALVPGLLREEVHVQ** | **SDPAGRLVIT** |
| HORVU6Hr1G070230.12 | 66 | 2.36e-19 | **EPADWVKINV** | **RQTKECFEIYALVPGLLREEVHVQ** | **SDPAGRLVIT** |
| HORVU6Hr1G070230.10 | 66 | 2.36e-19 | **EPADWVKINV** | **RQTKECFEIYALVPGLLREEVHVQ** | **SDPAGRLVIT** |
| HORVU6Hr1G070230.2 | 170 | 2.36e-19 | **EPADWVKINV** | **RQTKECFEIYALVPGLLREEVHVQ** | **SDPAGRLVIT** |
| HORVU6Hr1G070230.9 | 84 | 2.36e-19 | **EPADWVKINV** | **RQTKECFEIYALVPGLLREEVHVQ** | **SDPAGRLVIT** |
| HORVU4Hr1G063350.1 | 199 | 2.48e-16 | **GEMPRMPWDI** | **MEDDKEVKMRFDMPGLSREEVKVM** | **VEDDALVIRG** |
| HORVU4Hr1G072680.2 | 17 | 1.47e-15 | **YVDFVPSHDL** | **LEDNRKHTLVVNLPGFKKEHLRVQ** | **IDNYGLLRVS** |
| HORVU4Hr1G072680.3 | 48 | 1.47e-15 | **YVDFVPSHDL** | **LEDNRKHTLVVNLPGFKKEHLRVQ** | **IDNYGLLRVS** |
| HORVU3Hr1G020520.6 | 114 | 2.70e-14 | **RAMAATPADV** | **KELPGAYAFVVDMPGLGSGDINVQ** | **VEDERVLVIS** |
| HORVU5Hr1G061160.2 | 88 | 1.06e-13 | **PPDSKERWDI** | **KEEEDYVKLWFQVPGLSEDDLEIT** | **AGEDMLEIKR** |
| HORVU5Hr1G061160.3 | 88 | 1.06e-13 | **PPDSKERWDI** | **KEEEDYVKLWFQVPGLSEDDLEIT** | **AGEDMLEIKR** |
| HORVU5Hr1G061170.1 | 108 | 1.62e-13 | **VSPREERWQM** | **EEEADAVSMWFEVPGLSKEDLVVE** | **LDEDVLIIRR** |

Motif 3 located in the N terminal of HvHsp20s.


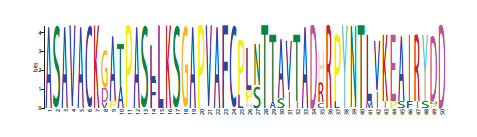


| Name | Start | *p*-value |  | Sites |  |
| --- | --- | --- | --- | --- | --- |
| HORVU7Hr1G036470.9 | 9 | 3.01e-62 | **EREERETM** | **ASAVACKGATPASLLKSGAPVAFCPLNTTAVTADHRPYNTLVKEAIRYDD** | **DYSGRDLVIP** |
| HORVU7Hr1G036470.3 | 38 | 3.01e-62 | **EKTDIERETM** | **ASAVACKGATPASLLKSGAPVAFCPLNTTAVTADHRPYNTLVKEAIRYDD** | **DYSGRDLVIP** |
| HORVU7Hr1G036500.5 | 24 | 3.01e-62 | **EKTDIERETM** | **ASAVACKGATPASLLKSGAPVAFCPLNTTAVTADHRPYNTLVKEAIRYDD** | **DDRNLVLPSF** |
| HORVU7Hr1G036500.7 | 2 | 3.01e-62 | **M** | **ASAVACKGATPASLLKSGAPVAFCPLNTTAVTADHRPYNTLVKEAIRYDD** | **DDRNLVLPSF** |
| HORVU7Hr1G036570.3 | 25 | 5.84e-62 | **EGERRERETM** | **ASAVACKGATPASFLKSGAPVAFCPLNTTSVTADHRPYNTLVKEAIRYDD** | **DDDYSGRHLV** |
| HORVU7Hr1G036570.2 | 30 | 5.84e-62 | **EGERRERETM** | **ASAVACKGATPASFLKSGAPVAFCPLNTTSVTADHRPYNTLVKEAIRYDD** | **DDDYSGRHLV** |
| HORVU7Hr1G036570.4 | 2 | 5.84e-62 | **M** | **ASAVACKGATPASFLKSGAPVAFCPLNTTSVTADHRPYNTLVKEAIRYDD** | **DDDYSGRHLV** |
| HORVU7Hr1G036470.21 | 27 | 1.38e-60 | **KREREERETM** | **ASAVACKGATPASFLKSGAPVAFCLPNTTAVTADHRPYNTLVKEAIRYDD** | **DDDYSGRHLV** |
| HORVU7Hr1G036470.20 | 31 | 1.38e-60 | **KREREERETM** | **ASAVACKGATPASFLKSGAPVAFCLPNTTAVTADHRPYNTLVKEAIRYDD** | **DDDYSGRHLV** |
| HORVU7Hr1G036470.12 | 12 | 2.79e-59 | **EKTDIERETM** | **ASAVACKGATPASFLKSGAPVAFCLPNTTAVTADHRLYNTLVKEAIRYDD** | **DDDYSGRHLV** |
| HORVU7Hr1G036470.6 | 23 | 2.79e-59 | **EKTDIERETM** | **ASAVACKGATPASFLKSGAPVAFCLPNTTAVTADHRLYNTLVKEAIRYDD** | **DDDYSGRHLV** |
| HORVU7Hr1G036470.2 | 29 | 1.41e-58 | **ESKERERETM** | **ASAVACKDAAPASLLKSGAPVAFCPLSTTAITADRRPYNTLIKEAIRYDD** | **DDDRNLVLPS** |
| HORVU7Hr1G036470.11 | 2 | 1.41e-58 | **M** | **ASAVACKDAAPASLLKSGAPVAFCPLSTTAITADRRPYNTLIKEAIRYDD** | **DDDRNLVLPS** |
| HORVU7Hr1G036500.1 | 2 | 1.41e-58 | **M** | **ASAVACKDAAPASLLKSGAPVAFCPLSTTAITADRRPYNTLIKEAIRYDD** | **DDDRNLVLPS** |
| HORVU7Hr1G036500.2 | 2 | 1.41e-58 | **M** | **ASAVACKDAAPASLLKSGAPVAFCPLSTTAITADRRPYNTLIKEAIRYDD** | **DDDRNLVLPS** |
| HORVU7Hr1G036500.4 | 45 | 1.41e-58 | **GKRERERETM** | **ASAVACKDAAPASLLKSGAPVAFCPLSTTAITADRRPYNTLIKEAIRYDD** | **DDDRNLVLPS** |
| HORVU7Hr1G036540.3 | 51 | 1.79e-57 | **KREKRERETM** | **ASAVACKVAAPASLLKSGAPVAFCPLSTTAVTADRRPYNTMVKEAIRYDD** | **DDDDYSGRHL** |
| HORVU7Hr1G036540.5 | 2 | 1.79e-57 | **M** | **ASAVACKVAAPASLLKSGAPVAFCPLSTTAVTADRRPYNTMVKEAIRYDD** | **DDDDYSGRHL** |
| HORVU7Hr1G036540.1 | 38 | 3.76e-50 | **KREREERETM** | **ASAVACKGPTPASFLKSGAPVAFCPHNTAAVIADCRPYNTLVIPSFISQD** | **VLDPLGAPTS** |
| HORVU7Hr1G036540.4 | 14 | 9.20e-50 | **KREREERETM** | **ASAVACKGPTPASFLKSGAPVAFCPHNTAAVIADCRPYNTLVIPSFISPD** | **VLDPLGAPTS** |

Motif 4 located between conserved region I and conserved region II of ACD domain of HvHsp20s.


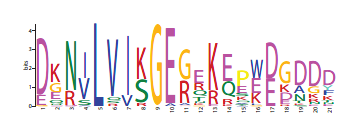


| Name | Start | *p*-value |  | Sites |  |
| --- | --- | --- | --- | --- | --- |
| HORVU7Hr1G036540.6 | 70 | 2.38e-27 | **LTKEHVQVRA** | **DKNILVIKGEGQKQPWDGDDD** | **SAVPRYNRRI** |
| HORVU7Hr1G036540.3 | 186 | 2.38e-27 | **LTKEHVQVRA** | **DKNILVIKGEGQKQPWDGDDD** | **SAVPRYNRRI** |
| HORVU7Hr1G036540.5 | 137 | 2.38e-27 | **LTKEHVQVRA** | **DKNILVIKGEGQKQPWDGDDD** | **SAVPRYNRRI** |
| HORVU7Hr1G036470.21 | 146 | 2.38e-27 | **LTKDHVQVCA** | **DKNILVIKGEGQKQPWDGDDD** | **SAVPRYNRRI** |
| HORVU7Hr1G036470.6 | 142 | 2.38e-27 | **LTKDHVQVCA** | **DKNILVIKGEGQKQPWDGDDD** | **SAVPRYNHRI** |
| HORVU7Hr1G036470.20 | 150 | 2.38e-27 | **LTKDHVQVCA** | **DKNILVIKGEGQKQPWDGDDD** | **SAVPRYNRRI** |
| HORVU7Hr1G036570.3 | 144 | 2.38e-27 | **LTKEHVQVRA** | **DKNILVIKGEGQKQPWDGDDD** | **SAVPRYNRRI** |
| HORVU7Hr1G036570.2 | 149 | 2.38e-27 | **LTKEHVQVRA** | **DKNILVIKGEGQKQPWDGDDD** | **SAVPRYNRRI** |
| HORVU7Hr1G036570.4 | 129 | 2.38e-27 | **LTKEHVQVRA** | **DKNILVIKGEGQKQPWDGDDD** | **SAVPRYNRRI** |
| HORVU7Hr1G036540.2 | 68 | 2.47e-25 | **LTKEHVQVRA** | **DKNILVIKGEGEKQPWDGDDN** | **SAVRRYNHRI** |
| HORVU7Hr1G036540.1 | 154 | 2.47e-25 | **LTKEHVQVRA** | **DKNILVIKGEGEKQPWDGDDN** | **SAVRRYNHRI** |
| HORVU7Hr1G036540.4 | 130 | 2.47e-25 | **LTKEHVQVRA** | **DKNILVIKGEGEKQPWDGDDN** | **SAVRRYNHRI** |
| HORVU7Hr1G036470.12 | 131 | 4.34e-25 | **LTKEHVQVRA** | **DKNILVIKGEGEKQPWDGDND** | **SAVPRYNHRI** |
| HORVU7Hr1G036470.14 | 109 | 4.34e-25 | **LTKEHVQVRA** | **DKNILVIKGEGEKQPWDGDND** | **SAVPRYNHRI** |
| HORVU7Hr1G036470.11 | 126 | 4.34e-25 | **LTKEHVQVRA** | **DKNILVIKGEGEKQPWDGDND** | **SAVPRYNHRI** |
| HORVU7Hr1G036470.2 | 153 | 4.25e-24 | **LTKEHVQVRA** | **DKNILVIKGEGEKRPWDGDDD** | **FAVPRYNHRI** |
| HORVU7Hr1G036470.10 | 169 | 4.25e-24 | **LTKEHVQVRA** | **DKNILVIKGEGEKRPWDGDDD** | **FAVPRYNHRI** |
| HORVU7Hr1G036470.9 | 141 | 4.25e-24 | **LTKEHVQVRA** | **DKNILVIKGEGEKRPWDGDDD** | **FAVPRYNHRI** |
| HORVU7Hr1G036470.3 | 170 | 4.25e-24 | **LTKEHVQVRA** | **DKNILVIKGEGEKRPWDGDDD** | **FAVPRYNHRI** |
| HORVU7Hr1G036500.7 | 132 | 1.64e-22 | **LTKEHVQVRA** | **DKNILVIKGEGEKQPLDGDDD** | **SAVPRYNRRI** |
| HORVU7Hr1G036500.2 | 126 | 1.64e-22 | **LTKEHVQVRA** | **DKNILVIKGEGEKQPLDGDDD** | **SAVPRYNRRI** |
| HORVU7Hr1G036500.5 | 154 | 6.42e-21 | **LTKEHVQVRA** | **GKNILVIKGEGEKRPWDGDDD** | **FAVSRYNHRI** |
| HORVU3Hr1G007500.4 | 140 | 3.90e-18 | **KKEEVKVEVE** | **DGNVLVVSGERTKEEEDKNDK** | **WHRVERSSGK** |
| HORVU3Hr1G007380.1 | 119 | 6.45e-18 | **KKEEVKVEVE** | **DGNVLVVSGERTKEKEDKNDK** | **WHRMERSSGK** |
| HORVU3Hr1G006530.1 | 134 | 6.45e-18 | **KKEEVKVEVE** | **DGNVLVVSGERTKEKEDKNDK** | **WHRVERSSGK** |
| HORVU2Hr1G120170.1 | 172 | 6.45e-18 | **KKEEVKVEVE** | **DGNVLVVSGERTKEKEDKNDK** | **WHRVERSSGK** |
| HORVU3Hr1G007500.1 | 127 | 6.45e-18 | **KKEEVKVEVE** | **DGNVLVVSGERTKEKEDKNDK** | **WHRVERSSGK** |
| HORVU3Hr1G007500.2 | 127 | 6.45e-18 | **KKEEVKVEVE** | **DGNVLVVSGERTKEKEDKNDK** | **WHRVERSSGK** |
| HORVU4Hr1G060720.1 | 74 | 7.12e-18 | **KKEEVKVEVE** | **DGNILQISGERNKEQEEKTDT** | **WHRVERSSGK** |
| HORVU4Hr1G060720.2 | 82 | 7.12e-18 | **KKEEVKVEVE** | **DGNILQISGERNKEQEEKTDT** | **WHRVERSSGK** |
| HORVU6Hr1G077710.3 | 204 | 1.06e-17 | **LGKEHVKVWA** | **EQNSLVIKGEGEKESEEEDGF** | **AAPRYSGRLE** |
| HORVU6Hr1G077710.5 | 63 | 1.06e-17 | **LGKEHVKVWA** | **EQNSLVIKGEGEKESEEEDGF** | **AAPRYSGRLE** |
| HORVU6Hr1G077710.2 | 176 | 1.06e-17 | **LGKEHVKVWA** | **EQNSLVIKGEGEKESEEEDGF** | **AAPRYSGRLE** |
| HORVU6Hr1G077710.4 | 98 | 1.06e-17 | **LGKEHVKVWA** | **EQNSLVIKGEGEKESEEEDGF** | **AAPRYSGRLE** |
| HORVU6Hr1G077710.6 | 135 | 1.06e-17 | **LGKEHVKVWA** | **EQNSLVIKGEGEKESEEEDGF** | **AAPRYSGRLE** |
| HORVU4Hr1G060760.1 | 74 | 2.27e-17 | **RKEEVKVEVD** | **DGNVLQISGERNKEQEEKTDT** | **WHHVERSSGR** |
| HORVU3Hr1G020500.1 | 150 | 6.90e-17 | **GSGDIKVQVE** | **DERVLVISGERRREEKEDAKY** | **VRMERRMGKM** |
| HORVU3Hr1G020520.4 | 85 | 6.90e-17 | **GSGDIKVQVE** | **DERVLVISGERRREEKEDAKY** | **VRMERRMGKM** |
| HORVU3Hr1G020520.6 | 140 | 6.90e-17 | **GSGDINVQVE** | **DERVLVISGERRREEKEDAKY** | **VRMERRMGKM** |
| HORVU3Hr1G020520.2 | 114 | 9.04e-17 | **GSGDIKVQVE** | **DERVLVISGERRREEKEDARY** | **VRMERRMGKM** |
| HORVU3Hr1G020520.1 | 82 | 9.04e-17 | **GSGDIKVQVE** | **DERVLVISGERRREEKEDARY** | **VRMERRMGKM** |
| HORVU3Hr1G020390.1 | 88 | 9.04e-17 | **GSGDIKVQVE** | **DERVLVISGERRREEKEDARY** | **LRMERRMGKL** |
| HORVU3Hr1G020490.2 | 112 | 1.18e-16 | **GSVDIKVQVE** | **DERVLVISGERRREEKEDAKF** | **VRMERRMGKL** |
| HORVU3Hr1G020490.3 | 113 | 1.18e-16 | **GSVDIKVQVE** | **DERVLVISGERRREEKEDAKF** | **VRMERRMGKL** |
| HORVU3Hr1G020520.5 | 85 | 6.06e-16 | **GSGDIKVQVE** | **DERVLVISGERGREEKEDARY** | **LRMERRMGKM** |
| HORVU0Hr1G020420.2 | 56 | 4.05e-11 | **GKDDVKVQVE** | **DGNVLSVRGAAKEKTKEGNEE** | **DAVWHVAERG** |
| HORVU0Hr1G020420.1 | 89 | 4.05e-11 | **GKDDVKVQVE** | **DGNVLSVRGAAKEKTKEGNEE** | **DAVWHVAERG** |
| HORVU4Hr1G063350.1 | 224 | 4.56e-11 | **LSREEVKVMV** | **EDDALVIRGEHKKEAGEGQGE** | **AAGGGDGWWK** |

Motif 5 located in conserved region II of ACD domain of HvHsp20s.


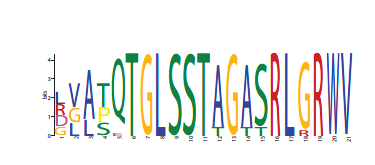


| Name | Start | *p*-value |  | Sites |  |
| --- | --- | --- | --- | --- | --- |
| HORVU7Hr1G036540.6 | 23 | 1.37e-25 | **SMARLLSLME** | **DVATQTGLSSTAGASRLGRWV** | **AKEDDGAVYL** |
| HORVU7Hr1G036540.3 | 139 | 1.37e-25 | **SMARLLSLME** | **DVATQTGLSSTAGASRLGRWV** | **AKEDDGAVYL** |
| HORVU7Hr1G036540.5 | 90 | 1.37e-25 | **SMARLLSLME** | **DVATQTGLSSTAGASRLGRWV** | **AKEDDGAVYL** |
| HORVU7Hr1G036470.3 | 123 | 5.32e-25 | **SMARLLSLME** | **GVATQTGLSSTAGASRLGRWV** | **AKEDDGAVYL** |
| HORVU7Hr1G036500.5 | 107 | 5.32e-25 | **SMARLLSLME** | **GVATQTGLSSTAGASRLGRWV** | **AKEEDGAVYL** |
| HORVU7Hr1G036470.21 | 99 | 2.65e-24 | **SFFSPDVLDP** | **LGAPQTGLSSTAGASRLGRWV** | **TKEDDGAVYL** |
| HORVU7Hr1G036470.12 | 84 | 2.65e-24 | **SFFSQDVLDP** | **LGAPQTGLSSTAGASRLGRWV** | **TKEDDGAVYL** |
| HORVU7Hr1G036470.6 | 95 | 2.65e-24 | **SFFSQDVLDP** | **LGAPQTGLSSTAGASRLGRWV** | **TKEDDGAVYL** |
| HORVU7Hr1G036470.20 | 103 | 2.65e-24 | **SFFSQDVLDP** | **LGAPQTGLSSTAGASRLGRWV** | **TKEDDGAVYL** |
| HORVU7Hr1G036470.2 | 106 | 6.54e-24 | **DPLGAPTGIA** | **RLLSQTGLSSTAGASRLGRWV** | **AKEDDGAVYL** |
| HORVU7Hr1G036470.14 | 62 | 6.54e-24 | **DPLGAPTGIA** | **RLLSQTGLSSTAGASRLGRWV** | **AKEDDGAVYL** |
| HORVU7Hr1G036470.11 | 79 | 6.54e-24 | **DPLGAPTGIA** | **RLLSQTGLSSTAGASRLGRWV** | **AKEDDGAVYL** |
| HORVU7Hr1G036500.1 | 79 | 6.54e-24 | **DPLGAPTGIA** | **RLLSQTGLSSTAGASRLGRWV** | **AKEDDGAVYL** |
| HORVU7Hr1G036500.2 | 79 | 6.54e-24 | **DPLGAPTGIA** | **RLLSQTGLSSTAGASRLGRWV** | **AKEDDGAVYL** |
| HORVU7Hr1G036500.4 | 122 | 6.54e-24 | **DPLGAPTGIA** | **RLLSQTGLSSTAGASRLGRWV** | **AKEDDGAVYL** |
| HORVU7Hr1G036570.3 | 97 | 8.04e-24 | **SFFSQGVLDP** | **LGAPQTGLSSTAGTSRLGRWV** | **TKEDDGAVYL** |
| HORVU7Hr1G036570.2 | 102 | 8.04e-24 | **SFFSQDVLDP** | **LGAPQTGLSSTAGTSRLGRWV** | **TKEDDGAVYL** |
| HORVU7Hr1G036570.4 | 82 | 8.04e-24 | **DVDSADVLDP** | **LGAPQTGLSSTAGTSRLGRWV** | **TKEDDGAVYL** |
| HORVU7Hr1G036540.2 | 21 | 9.76e-24 | **SMARLLSRVD** | **DVATQTGLSSTTGATRLGRWV** | **AKEDDGAVYL** |
| HORVU7Hr1G036540.1 | 107 | 9.76e-24 | **SMARLLSRVD** | **DVATQTGLSSTTGATRLGRWV** | **AKEDDGAVYL** |
| HORVU7Hr1G036540.4 | 83 | 9.76e-24 | **SMARLLSRVD** | **DVATQTGLSSTTGATRLGRWV** | **AKEDDGAVYL** |
| HORVU7Hr1G036470.10 | 122 | 1.38e-23 | **SMARLLSLME** | **GVATQTGLSSTAGASRLRRWV** | **AKEDDGAVYL** |
| HORVU7Hr1G036470.9 | 94 | 1.38e-23 | **SMARLLSLME** | **GVATQTGLSSTAGASRLRRWV** | **AKEDDGAVYL** |
| HORVU7Hr1G036500.7 | 85 | 3.21e-23 | **SMARLPSLME** | **GVATETGLSSTAGASRLGRWV** | **AKEDDGAVYL** |

Motif 6 located in conserved region I of ACD domain of HvHsp20s.


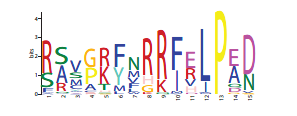


| Name | Start | *p*-value |  | Sites |  |
| --- | --- | --- | --- | --- | --- |
| HORVU7Hr1G036540.6 | 91 | 3.68e-15 | **QKQPWDGDDD** | **SAVPRYNRRIELPAD** | **AYKMDKIKAE** |
| HORVU7Hr1G036540.3 | 207 | 3.68e-15 | **QKQPWDGDDD** | **SAVPRYNRRIELPAD** | **AYKMDKIKAE** |
| HORVU7Hr1G036540.5 | 158 | 3.68e-15 | **QKQPWDGDDD** | **SAVPRYNRRIELPAD** | **AYKMDKIKAE** |
| HORVU7Hr1G036470.21 | 167 | 3.68e-15 | **QKQPWDGDDD** | **SAVPRYNRRIELPAD** | **AYKMDKIKAE** |
| HORVU7Hr1G036470.20 | 171 | 3.68e-15 | **QKQPWDGDDD** | **SAVPRYNRRIELPAD** | **AYKMDKIKAE** |
| HORVU7Hr1G036570.3 | 165 | 3.68e-15 | **QKQPWDGDDD** | **SAVPRYNRRIELPAD** | **AYKMDKIKAE** |
| HORVU7Hr1G036570.2 | 170 | 3.68e-15 | **QKQPWDGDDD** | **SAVPRYNRRIELPAD** | **AYKMDKIKAE** |
| HORVU7Hr1G036570.4 | 150 | 3.68e-15 | **QKQPWDGDDD** | **SAVPRYNRRIELPAD** | **AYKMDKIKAE** |
| HORVU7Hr1G036500.7 | 153 | 3.68e-15 | **EKQPLDGDDD** | **SAVPRYNRRIELPAD** | **AYKMDKIKAE** |
| HORVU7Hr1G036500.2 | 147 | 3.68e-15 | **EKQPLDGDDD** | **SAVPRYNRRIELPAD** | **AYKMDKIKAE** |
| HORVU7Hr1G036470.12 | 152 | 1.72e-14 | **EKQPWDGDND** | **SAVPRYNHRIEIPAD** | **AYKMDKIKAE** |
| HORVU7Hr1G036470.6 | 163 | 1.72e-14 | **QKQPWDGDDD** | **SAVPRYNHRIEIPAD** | **AYKMDKIKAE** |
| HORVU7Hr1G036470.14 | 130 | 1.72e-14 | **EKQPWDGDND** | **SAVPRYNHRIEIPAD** | **AYKMDKIKAE** |
| HORVU7Hr1G036470.11 | 147 | 1.72e-14 | **EKQPWDGDND** | **SAVPRYNHRIEIPAD** | **AYKMDKIKAE** |
| HORVU3Hr1G007380.1 | 145 | 2.26e-14 | **DKNDKWHRME** | **RSSGKFVRRFRLPED** | **AKVEEVKAGL** |
| HORVU3Hr1G006530.1 | 160 | 2.26e-14 | **DKNDKWHRVE** | **RSSGKFVRRFRLPED** | **AKVEEVKAGL** |
| HORVU2Hr1G120170.1 | 198 | 2.26e-14 | **DKNDKWHRVE** | **RSSGKFVRRFRLPED** | **AKVEEVKAGL** |
| HORVU3Hr1G007500.1 | 153 | 2.26e-14 | **DKNDKWHRVE** | **RSSGKFVRRFRLPED** | **AKVEEVKAGL** |
| HORVU3Hr1G007500.2 | 153 | 2.26e-14 | **DKNDKWHRVE** | **RSSGKFVRRFRLPED** | **AKVEEVKAGL** |
| HORVU3Hr1G007500.4 | 166 | 2.26e-14 | **DKNDKWHRVE** | **RSSGKFVRRFRLPED** | **AKVEEVKAGL** |
| HORVU3Hr1G006930.1 | 103 | 2.59e-14 | **AKNETRHHVE** | **RSCATFFGRFHLPED** | **AALGRVRAAM** |
| HORVU3Hr1G006930.2 | 103 | 2.59e-14 | **AKNETRHHVE** | **RSCATFFGRFHLPED** | **AALGRVRAAM** |
| HORVU4Hr1G060720.1 | 100 | 2.59e-14 | **EKTDTWHRVE** | **RSSGKFLRRFRLPEN** | **AKAEQVKASM** |
| HORVU4Hr1G060720.2 | 108 | 2.59e-14 | **EKTDTWHRVE** | **RSSGKFLRRFRLPEN** | **AKAEQVKASM** |
| HORVU7Hr1G036470.2 | 174 | 2.95e-14 | **EKRPWDGDDD** | **FAVPRYNHRIEIPAD** | **AYKMDKIKAE** |
| HORVU7Hr1G036470.10 | 190 | 2.95e-14 | **EKRPWDGDDD** | **FAVPRYNHRIEIPAD** | **AYKMDKIKAE** |
| HORVU7Hr1G036470.9 | 162 | 2.95e-14 | **EKRPWDGDDD** | **FAVPRYNHRIEIPAD** | **AYKMDKIKAE** |
| HORVU7Hr1G036470.3 | 191 | 2.95e-14 | **EKRPWDGDDD** | **FAVPRYNHRIEIPAD** | **AYKMDKIKAE** |
| HORVU3Hr1G006940.5 | 103 | 4.31e-14 | **AKSETRHHVE** | **RSCATFFGRFHLPQD** | **AALGQVRAAM** |
| HORVU3Hr1G006940.3 | 124 | 4.31e-14 | **AKSETRHHVE** | **RSCATFFGRFHLPQD** | **AALGQVRAAM** |
| HORVU3Hr1G006940.2 | 103 | 4.31e-14 | **AKSETRHHVE** | **RSCATFFGRFHLPQD** | **AALGQVEPAE** |
| HORVU3Hr1G006940.6 | 123 | 4.31e-14 | **AKSETRHHVE** | **RSCATFFGRFHLPQD** | **AALGQVRAAM** |
| HORVU3Hr1G006940.4 | 103 | 4.31e-14 | **AKSETRHHVE** | **RSCATFFGRFHLPQD** | **AALGQVRAAM** |
| HORVU3Hr1G020500.1 | 175 | 7.85e-14 | **KEDAKYVRME** | **RRMGKMMRKFVLPEN** | **ADMEKIAAAC** |
| HORVU3Hr1G020520.4 | 110 | 7.85e-14 | **KEDAKYVRME** | **RRMGKMMRKFVLPEN** | **ADMEKISAAC** |
| HORVU3Hr1G020520.2 | 139 | 7.85e-14 | **KEDARYVRME** | **RRMGKMMRKFVLPEN** | **ADMEKISAAC** |
| HORVU3Hr1G020520.6 | 165 | 7.85e-14 | **KEDAKYVRME** | **RRMGKMMRKFVLPEN** | **ADMEKISAAC** |
| HORVU3Hr1G020520.1 | 107 | 7.85e-14 | **KEDARYVRME** | **RRMGKMMRKFVLPEN** | **ADMEKISAAC** |
| HORVU4Hr1G060760.1 | 100 | 8.79e-14 | **EKTDTWHHVE** | **RSSGRFLRRFRLPDN** | **ARAEEIKAAM** |
| HORVU3Hr1G020520.5 | 110 | 2.31e-13 | **KEDARYLRME** | **RRMGKMMRKFVLPDN** | **ADMEKISAAC** |
| HORVU3Hr1G020490.2 | 137 | 5.08e-13 | **KEDAKFVRME** | **RRMGKLMRKFVLPEN** | **ADMEKISAAC** |
| HORVU3Hr1G020490.3 | 138 | 5.08e-13 | **KEDAKFVRME** | **RRMGKLMRKFVLPEN** | **ADMEKISAAC** |
| HORVU3Hr1G020390.1 | 113 | 2.78e-11 | **KEDARYLRME** | **RRMGKLMRKFVVPDN** | **ADTEKISAVC** |
| HORVU4Hr1G015170.1 | 166 | 5.47e-11 | **KEGERWHRAE** | **RAAGRFWRRFRMPAG** | **ADVERVTARL** |
| HORVU1Hr1G035950.2 | 36 | 5.84e-10 | **ISGERAVNGG** | **RQWCHFLKRFDLPGV** | **CDAAAIKVQL** |
| HORVU1Hr1G035950.1 | 121 | 5.84e-10 | **ISGERAVNGG** | **RQWCHFLKRFDLPGV** | **CDAAAIKVQL** |
| HORVU6Hr1G082360.1 | 230 | 1.12e-9 | **DCRYIRLERR** | **ASPRSFVRKFRLPED** | **ADAGAVAARC** |
| HORVU1Hr1G066530.2 | 140 | 1.74e-9 | **ADGRDWRAGR** | **WWEHGFVRRVELPED** | **ADGGRVEAYF** |

Motif 7 located in conserved region II of ACD domain of HvHsp20s.


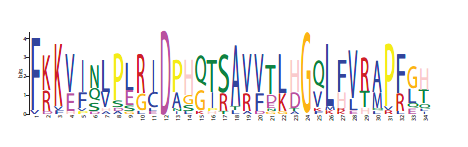


| Name | Start | *p*-value |  | Sites |  |
| --- | --- | --- | --- | --- | --- |
| HORVU6Hr1G070230.11 | 94 | 4.86e-43 | **QPDNPWGITA** | **FKKVINLPLRIDPHQTSAVVTLHGQLFVRAPFGH** | **PDMF** |
| HORVU6Hr1G070230.12 | 114 | 4.86e-43 | **QPDNPWGITA** | **FKKVINLPLRIDPHQTSAVVTLHGQLFVRAPFGH** | **PDM** |
| HORVU6Hr1G070230.10 | 114 | 4.86e-43 | **QPDNPWGITA** | **FKKVINLPLRIDPHQTSAVVTLHGQLFVRAPFGH** | **PDM** |
| HORVU6Hr1G070230.2 | 218 | 4.86e-43 | **QPDNPWGITA** | **FKKVINLPLRIDPHQTSAVVTLHGQLFVRAPFGH** | **PDM** |
| HORVU6Hr1G070230.9 | 132 | 4.86e-43 | **QPDNPWGITA** | **FKKVINLPLRIDPHQTSAVVTLHGQLFVRAPFGH** | **PDM** |
| HORVU4Hr1G072700.1 | 93 | 9.96e-29 | **RPLEGGQWRR** | **FRKEFQVPEGCDAGGIRARFDKDGVLHVTMPRLT** | **PLDLEDDPKA** |
| HORVU4Hr1G072680.2 | 63 | 5.32e-28 | **RPLEGGQWSR** | **FRKEFQVPEGCDAGGIRARFDKDGVLHLTMPRLT** | **PLELDPKAAA** |
| HORVU4Hr1G072680.3 | 94 | 5.32e-28 | **RPLEGGQWSR** | **FRKEFQVPEGCDAGGIRARFDKDGVLHLTMPRLT** | **PLELDPKAAA** |
| HORVU4Hr1G072770.1 | 36 | 1.82e-27 | **RPLEDGQWSR** | **FRKEFQVSEGCDASGIRARFEKDGVLHVTMPRLT** | **PLEDDPKAAA** |
| HORVU4Hr1G072770.2 | 92 | 1.82e-27 | **RPLEDGQWSR** | **FRKEFQVSEGCDASGIRARFEKDGVLHVTMPRLT** | **PLEDDPKAAA** |
| HORVU4Hr1G072700.1 | 38 | 2.38e-14 | **QLSMAAERTY** | **VDFVPSHDLLEDNHKHTLVVNLTGFKKEHLRLQI** | **DKSGRLRVSG** |
| HORVU3Hr1G051000.3 | 20 | 1.84e-13 | **QPPRGLKKEE** | **FRVHVDAAGRLDILGPSTADGGAGKMRLHQVFQL** | **PATSDLDAIT** |

Motif 8 located in conserved region II of ACD domain of HvHsp20s.


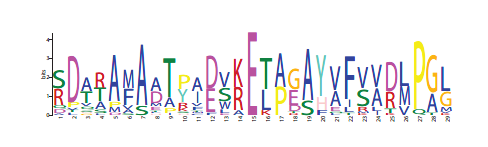


|  | Name | Start | *p*-value |  | Sites |  |
| --- | --- | --- | --- | --- | --- | --- |
|  | HORVU3Hr1G006940.5 | 36 | 8.47e-29 | **ASGGADAWLA** | **SDTTAFADTYIESRETAEAYVFSARLPAG** | **VTKEEVKVEV** |
|  | HORVU3Hr1G006940.3 | 57 | 8.47e-29 | **ASGGADAWLA** | **SDTTAFADTYIESRETAEAYVFSARLPAG** | **VTKEEVKVEV** |
|  | HORVU3Hr1G006940.2 | 36 | 8.47e-29 | **ASGGADAWLA** | **SDTTAFADTYIESRETAEAYVFSARLPAG** | **VTKEEVKVEV** |
|  | HORVU3Hr1G006940.6 | 56 | 8.47e-29 | **ASGGADAWLA** | **SDTTAFADTYIESRETAEAYVFSARLPAG** | **VTKEEVKVEV** |
|  | HORVU3Hr1G006940.4 | 36 | 8.47e-29 | **ASGGADAWLA** | **SDTTAFADTYIESRETAEAYVFSARLPAG** | **VTKEEVKVEV** |
|  | HORVU3Hr1G006930.1 | 36 | 8.47e-29 | **ASGGADAWLA** | **SDTTAFADTYIESRETAEAYVFSARLPAG** | **VTKEEVKVEV** |
|  | HORVU3Hr1G006930.2 | 36 | 8.47e-29 | **ASGGADAWLA** | **SDTTAFADTYIESRETAEAYVFSARLPAG** | **VTKEEVKVEV** |
|  | HORVU3Hr1G020500.1 | 111 | 3.45e-28 | **EKQGPTRAYV** | **RDARAMAATPADVKELPGAYAFVVDMPGL** | **GSGDIKVQVE** |
|  | HORVU3Hr1G020520.4 | 46 | 3.45e-28 | **EKQGPTRTYV** | **RDARAMAATPADVKELPGAYAFVVDMPGL** | **GSGDIKVQVE** |
|  | HORVU3Hr1G020520.2 | 75 | 3.45e-28 | **EKQGPTRAYV** | **RDARAMAATPADVKELPGAYAFVVDMPGL** | **GSGDIKVQVE** |
|  | HORVU3Hr1G020520.1 | 43 | 3.45e-28 | **EKQGPTRAYV** | **RDARAMAATPADVKELPGAYAFVVDMPGL** | **GSGDIKVQVE** |
|  | HORVU3Hr1G020520.5 | 46 | 3.45e-28 | **EKQGPTRTYV** | **RDARAMAATPADVKELPGAYAFVVDMPGL** | **GSGDIKVQVE** |
|  | HORVU3Hr1G020390.1 | 49 | 2.45e-27 | **EKQGPTRAYV** | **RDARAMAATPADVKELPGAYLFVVDMPGL** | **GSGDIKVQVE** |
|  | HORVU3Hr1G020490.2 | 73 | 2.00e-26 | **EKQGPTRAYV** | **RDARAMAATPADVKELPGAFAFVVDMPGL** | **GSVDIKVQVE** |
|  | HORVU3Hr1G020490.3 | 74 | 2.00e-26 | **EKQGPTRAYV** | **RDARAMAATPADVKELPGAFAFVVDMPGL** | **GSVDIKVQVE** |
|  | HORVU4Hr1G015170.1 | 101 | 3.83e-18 | **RPASAGDPAA** | **SVSSPMALARCDWKETPDAHVISLDVPGV** | **RRDDVKVEVE** |
|  | HORVU6Hr1G015130.1 | 60 | 7.31e-17 | **AISLKAFGGP** | **ALGLPFSTASMDWKETPTAHVFMADVPGL** | **RREEVKVEVE** |
|  | HORVU1Hr1G094450.1 | 11 | 9.27e-16 | **MAATGARQQQ** | **QPQAAAAEPKFELAEKAGSYVLRITLQGL** | **RKDDFRVQVD** |
|  | HORVU1Hr1G094450.2 | 11 | 9.27e-16 | **MAATGARQQQ** | **QPQAAAAEPKFELAEKAGSYVLRITLQGL** | **RKDDFRVQVD** |
|  | HORVU4Hr1G015170.1 | 4 | 1.99e-12 | **KES** | **STHSDSAHIHTFEKETARSHQTKLKLPTK** | **QVAMAAVSKK** |

Motif 9 located in the N terminal of HvHsp20s.


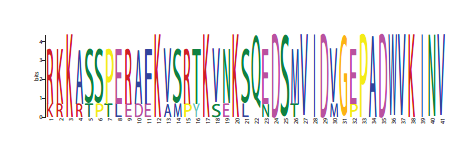


| Name | Start | *p*-value |  | Sites |  |
| --- | --- | --- | --- | --- | --- |
| HORVU6Hr1G070230.11 | 5 | 5.42e-51 | **GVLK** | **RKKASSPERAFKVSRTKVNKSQEDSMVIDVGEPADWVKINV** | **RQTKECFEIY** |
| HORVU6Hr1G070230.12 | 25 | 5.42e-51 | **YLSGISGVLK** | **RKKASSPERAFKVSRTKVNKSQEDSMVIDVGEPADWVKINV** | **RQTKECFEIY** |
| HORVU6Hr1G070230.10 | 25 | 5.42e-51 | **KNLKGFGVLK** | **RKKASSPERAFKVSRTKVNKSQEDSMVIDVGEPADWVKINV** | **RQTKECFEIY** |
| HORVU6Hr1G070230.2 | 129 | 5.42e-51 | **KNLKGFGVLK** | **RKKASSPERAFKVSRTKVNKSQEDSMVIDVGEPADWVKINV** | **RQTKECFEIY** |
| HORVU6Hr1G070230.9 | 43 | 5.42e-51 | **KNLKGFGVLK** | **RKKASSPERAFKVSRTKVNKSQEDSMVIDVGEPADWVKINV** | **RQTKECFEIY** |

Motif 10 located in C-terminal of HvHsp20s.


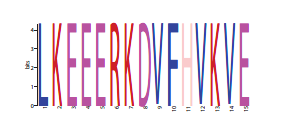


| Name | Start | *p*-value |  | Sites |  |
| --- | --- | --- | --- | --- | --- |
| HORVU7Hr1G036540.6 | 128 | 1.47e-18 | **NGVLWVTLLK** | **LKEEERKDVFHVKVE** |  |
| HORVU7Hr1G036540.3 | 244 | 1.47e-18 | **NGVLWVTLLK** | **LKEEERKDVFHVKVE** |  |
| HORVU7Hr1G036540.2 | 126 | 1.47e-18 | **NGVLWVTLIK** | **LKEEERKDVFHVKVE** |  |
| HORVU7Hr1G036540.1 | 212 | 1.47e-18 | **NGVLWVTLIK** | **LKEEERKDVFHVKVE** |  |
| HORVU7Hr1G036540.5 | 195 | 1.47e-18 | **NGVLWVTLLK** | **LKEEERKDVFHVKVE** |  |
| HORVU7Hr1G036540.4 | 188 | 1.47e-18 | **NGVLWVTLIK** | **LKEEERKDVFHVKVE** |  |
| HORVU7Hr1G036470.21 | 204 | 1.47e-18 | **NGVLWVTLLK** | **LKEEERKDVFHVKVE** |  |
| HORVU7Hr1G036470.2 | 211 | 1.47e-18 | **NGMLWVTLLK** | **LKEEERKDVFHVKVE** |  |
| HORVU7Hr1G036470.6 | 200 | 1.47e-18 | **NGMLWVTLLK** | **LKEEERKDVFHVKVE** |  |
| HORVU7Hr1G036470.20 | 208 | 1.47e-18 | **NGVLWVTLLK** | **LKEEERKDVFHVKVE** |  |
| HORVU7Hr1G036470.10 | 227 | 1.47e-18 | **NGMLWVTLLK** | **LKEEERKDVFHVKVE** |  |
| HORVU7Hr1G036470.9 | 199 | 1.47e-18 | **NGMLWVTLLK** | **LKEEERKDVFHVKVE** |  |
| HORVU7Hr1G036470.3 | 228 | 1.47e-18 | **NGMLWVTLLK** | **LKEEERKDVFHVKVE** |  |
| HORVU7Hr1G036570.3 | 202 | 1.47e-18 | **NGVLWVTLLK** | **LKEEERKDVFHVKVE** |  |
| HORVU7Hr1G036570.2 | 207 | 1.47e-18 | **NGVLWVTLLK** | **LKEEERKDVFHVKVE** |  |
| HORVU7Hr1G036570.4 | 187 | 1.47e-18 | **NGVLWVTLLK** | **LKEEERKDVFHVKVE** |  |
| HORVU7Hr1G036500.7 | 190 | 1.47e-18 | **NGVLWVTLLK** | **LKEEERKDVFHVKVE** |  |
| HORVU7Hr1G036500.2 | 184 | 1.47e-18 | **NGVLWVTLLK** | **LKEEERKDVFHVKVE** |  |
