# Supplementary material for: Genome-wide identification and expression profile analysis of the Hsp20 gene family in Barley (Hordeum vulgare L.)
Source: PeerJ. 2019 May 3;7:e6832. doi: 10.7717/peerj.6832 (PMC6501772; doi:10.7717/peerj.6832)
Supplement: Supplemental Information 5 [file peerj-07-6832-s005.docx]

**Table S3** **The number and composition of promoter elements of each *HvHsp20* gene.**

|  | MBS | CCAAT-box | MYB | ABRE | G-box | TC-rich repeats | W-box | LTR | DRE_core |
| --- | --- | --- | --- | --- | --- | --- | --- | --- | --- |
| HORVU0Hr1G020420 | 3 |  |  | 2 | 2 | 2 | 4 |  |  |
| HORVU1Hr1G035950 | 1 | 1 |  | 8 | 7 | 1 | 1 | 1 | 2 |
| HORVU1Hr1G066530 |  | 1 |  | 8 | 6 |  | 1 |  |  |
| HORVU1Hr1G094440 |  |  |  |  | 2 |  |  | 1 | 3 |
| HORVU1Hr1G094450 | 1 |  |  | 4 | 3 |  | 1 |  |  |
| HORVU1Hr1G094510 | 1 |  |  | 9 | 4 |  | 1 |  | 1 |
| HORVU2Hr1G046370 | 1 |  |  | 5 | 3 |  |  |  | 1 |
| HORVU2Hr1G058170 |  |  |  |  | 1 |  | 1 | 1 | 1 |
| HORVU3Hr1G006530 |  | 1 |  | 6 | 2 |  | 1 |  |  |
| HORVU3Hr1G006930 |  | 2 | 1 | 8 | 7 | 1 |  | 1 | 1 |
| HORVU3Hr1G006940 | 1 |  |  | 4 | 3 | 1 | 1 |  | 2 |
| HORVU3Hr1G007380 |  |  | 2 |  |  |  | 1 |  | 1 |
| HORVU3Hr1G007500 |  | 1 | 1 | 2 | 3 |  |  | 1 |  |
| HORVU3Hr1G020390 |  |  | 2 |  |  |  |  | 3 |  |
| HORVU3Hr1G020490 |  | 1 | 1 | 1 | 1 | 1 |  | 1 | 1 |
| HORVU3Hr1G020500 |  |  |  | 2 | 1 |  |  |  |  |
| HORVU3Hr1G020520 | 1 |  |  | 1 | 2 |  | 1 | 1 |  |
| HORVU3Hr1G051000 | 2 |  |  | 1 | 1 |  |  |  |  |
| HORVU4Hr1G002280 |  |  |  | 1 | 1 |  | 1 |  |  |
| HORVU4Hr1G002290 | 1 |  |  |  |  |  |  | 1 |  |
| HORVU4Hr1G015170 |  |  |  | 3 | 4 |  | 2 |  | 1 |
| HORVU4Hr1G060720 |  |  |  | 1 |  | 1 |  |  | 1 |
| HORVU4Hr1G060760 |  |  |  | 2 | 2 | 1 |  | 2 |  |
| HORVU4Hr1G063350 |  | 2 |  | 2 | 2 | 1 |  |  |  |
| HORVU4Hr1G072680 | 1 |  |  | 7 | 7 |  |  |  |  |
| HORVU4Hr1G072700 | 1 |  |  | 3 | 3 | 1 | 1 | 1 |  |
| HORVU4Hr1G072770 | 1 |  |  | 1 | 2 |  |  |  | 2 |
| HORVU5Hr1G006670 | 1 | 3 |  | 1 | 1 |  | 2 | 1 | 1 |
| HORVU5Hr1G061160 |  | 1 |  | 1 | 1 |  |  |  | 1 |
| HORVU5Hr1G061170 | 1 | 2 |  | 3 | 4 | 1 |  |  | 1 |
| HORVU6Hr1G015130 |  |  |  |  |  |  |  |  | 1 |
| HORVU6Hr1G070230 | 1 |  |  | 5 | 4 | 1 | 1 |  | 2 |
| HORVU6Hr1G077710 | 1 |  |  |  |  |  |  |  | 1 |
| HORVU6Hr1G082360 | 1 |  |  |  |  | 2 |  |  |  |
| HORVU7Hr1G036470 |  |  |  |  |  |  | 1 |  |  |
| HORVU7Hr1G036500 |  |  |  |  | 1 |  |  | 1 |  |
| HORVU7Hr1G036540 | 2 |  |  | 3 | 5 |  |  |  | 5 |
| HORVU7Hr1G036570 |  | 1 |  | 1 |  |  |  | 1 | 1 |
